# Shared care for children with cancer in India through social and healthcare partnerships during the COVID‐19 pandemic

**DOI:** 10.1002/cnr2.1486

**Published:** 2021-06-27

**Authors:** Amita Mahajan, Ramandeep Singh Arora, Puneet Kaur Sahi, Sunil Gomber, Nita Radhakrishnan, Basab Bagchi, Prachi Jain, Arvind Kumar, Avinash Singh, Haresh Gupta, Sonal Sharma, Nasim Ahamad, Poonam Bagai, Arvind Kumar

**Affiliations:** ^1^ Department of Pediatrics Oncology and Hematology Indraprastha Apollo Hospital Delhi India; ^2^ Department of Pediatric Oncology Max Super Specialty Hospital, Saket Delhi India; ^3^ Department of Pediatrics Maulana Azad Medical College and Lok Nayak Hospital Delhi India; ^4^ Department of Pediatric University College of Medical Sciences and Guru Teg Bahadur Hospital Delhi India; ^5^ Department of Paediatric Hemato‐Oncology Super Speciality Paediatric Hospital and Post Graduate Teaching Institute Noida Uttar Pradesh India; ^6^ Department of Medical Oncology & Haematology All India Institute of Medical Sciences Patna Bihar India; ^7^ Department of Pediatric Oncology Max Super Specialty Hospital, Vaishali Delhi India; ^8^ Department of Hemato‐Oncologist Buddha Cancer Centre Patna Bihar India; ^9^ Department of Hemato‐Oncology Paras HMRI Hospital Patna Bihar India; ^10^ Department of Medical Projects and Social Support Program Cankids Kidscan Delhi India

**Keywords:** childhood cancer, COVID‐19, non‐government organization, shared care

## Abstract

**Background:**

The COVID pandemic posed a challenge for the tertiary centers to continue treatment. Some tertiary centers were designated as COVID‐only hospitals, making it difficult for existing childhood cancer patients to continue their treatment at those centres. The need for shared care in childhood cancer was perceived by Cankids and its partnering childhood cancer‐treating centers in North and East India.

**Aim:**

We aim to show how Cankids upscaled its shared care model to ensure that COVID designated hospitals connected with other hospitals who have to continue to provide care to childhood cancer patients in the pandemic and thus ensured the continuation of treatment for these patients.

**Methods and result:**

The need assessment of the beneficiaries was done in discussion with the hospital of origin and destination hospital. The need for shared care was also discussed with the families and consent was taken before shifting their children. Cankids with the help of advisors identified cases of high risk that need immediate attention, proactive regular monitoring, and help in care planning with the perspective and recommendation of the multiple providers. The shared care unit came forward with reasonable and discounted packages for treatment.

There was a total of five hospitals requiring shared care, and 55 children were supported from April to November 2020. The median age was 8 years and their hospital of origin are in Bihar, Uttar Pradesh, West Bengal, and Delhi. The expenditure on the treatment of the 55 patients was INR 61 61 636 ($ 84 843), with a median of INR 41765 (IQR 19491–174 129) on each patient. Total 291 trips for the transport were arranged and all the patients combined stayed 174 days at Cankids accommodation facility.

**Conclusion:**

The shared care helped the patients access standard treatment and reduce the financial burden.

## 1 INTRODUCTION

The world is currently experiencing the COVID‐19 pandemic.^1^ The first patient with SARS‐CoV‐2 infection from India was reported from Kerala at the end of January 2020.^2^ Following the WHO declaration of the global pandemic on the 11th of March 2020, on the 24th of March 2020, the Government of India announced a national lockdown.^3^ Many tertiary centers that were providing specialized treatment for various illnesses designated as dedicated COVID hospitals.^4^ Consequently, children with cancer suddenly found themselves without their treatment facility. There was no also governmental or institutional directive on an alternative for these patients.

Cankids (www.cankidsindia.org) is a civil society organization working since the year 2004 for change for childhood cancer in India and today supports more than 11 000 patients every year by enabling access to standard care, ensuring holistic care, quality of life, and improving survival of these patients.^5^ As of 2020, Cankids has a presence in 113 hospitals which includes 61 hospitals across North and East India. And so Cankids was well placed to reduce the interruption in treatment of these children during the pandemic and the lockdown. The extraordinary situation created by COVID‐19 created an increased need for shared care by finding an alternative facility so that these children with cancer could continue treatment uninterrupted.

Facilitating shared care between two or more tertiary centers as a pediatric oncology outreach clinic has been a small and more recent part of the Cankids portfolio even before the COVID‐19 pandemic.^6^, ^7^ Shared care between Dayanand Medical College and Hospital (DMCH), Ludhiana, and Advanced Cancer Institute (ACI), Bhatinda, Punjab in North India which are approximately 150 km distance from each other is one such example. DMCH is a private sector hospital with the facility of pediatric oncologists besides all other components to provide multidisciplinary care to children with cancer. ACI Bhatinda has a team of radiation oncologist, pathologist, and onco‐surgery but does not have a dedicated pediatric oncologist. ACI Bhatinda provides surgery and radiation at low cost. The services such as chemotherapy, transfusion support, and intensive care services are provided at the Department of Pediatrics, DMCH. Transport facilities, chemotherapy drugs, accommodation, and holistic care are provided by Cankids. In the year 2018–19, a total of 67 patients benefitted through shared care.^6^ Another example of shared care is in Max Super Specialty Hospital, Delhi where Cankids facilitated specialized surgery for children with bone and soft tissue sarcoma. Patients receiving chemotherapy at their local pediatric oncology centres were brought to Delhi and 16 patients benefitted under this model.^8^

With this context, we aimed to show how Cankids upscaled its shared care model to ensure that COVID designated hospitals connected with other hospitals to continue providing care to childhood cancer patients in the pandemic and thus ensured the continuation of treatment for these patients.

## 2 METHODS

### 2.1 Setting

Hospital of Origin – Hospitals in India that stopped providing service to childhood cancer patients as a result of the COVID 19 pandemic as they were designated COVID 19 hospitals.

Destination hospital – Hospitals usually in the same geographic area who were already providing care to children with cancer and were able to accommodate additional patients and ensure uninterrupted treatment for the period of the pandemic.

Facilitation By – Cankids, a community‐based organization working across India.

### 2.2 Time‐period

For varying periods starting from April 2020 till January 2021.

### 2.3 Inclusion criteria

Patients up to 19 years of age with a confirmed diagnosis of cancer, who were undergoing treatment at the hospitals of origin which were closed for service due to COVID 19 pandemic, and who needed to continue active treatment needing inpatient or daycare admissions.

### 2.4 Exclusion criteria

Those who needed only follow‐up and had completed their treatment for example, survivors.

### 2.5 Linking hospitals

The hospitals of origin approached Cankids after being declared as a COVID designated center. The treating doctors of those hospitals and the social support team of the Cankids identified the patients who needed inpatient or day‐care admissions for active treatment. Cankids emergency response committee identified potential destination hospitals, wherever possible, in the same city or nearby city as the hospital of origin. Discussions were done with the destination hospital treating team, their management, and the Cankids emergency response committee to assess the feasibility, negotiate the price, and eventually have a memorandum of understanding. The resources for diagnosis and treatment at the destination hospitals were arranged from the existing budget of Cankids allocated for shared care as well as additional funding raised through online crowd‐funding platforms.

### 2.6 Enabling transfer

The parents/ guardians of the patients were informed by the treating team of the hospitals of origin as well as by the social support team of Cankids. The information about the need for shifting was communicated with them. Their approval was taken before they were shifted to destination hospitals. At the destination hospitals, they consented to treatment. There was regular communication between the treating team at the hospital of origin and the destination hospital on the relevant medical issues about each patient.

As the lockdown led to travel restrictions, particularly for inter‐state travel, special permissions from the district/ state administration were obtained by the management team of the Cankids. Standard COVID‐19 precautions as per the government of India were followed during transporting patients and their families. Decontamination of the ambulance was performed every time a patient was transported in the ambulance. All horizontal, vertical, and contact surfaces were disinfected with a cotton cloth saturated with a 1% sodium hypochlorite solution. Change of cotton mop and water containing disinfectant was ensured after each cleaning cycle. The biomedical waste generated (including PPE) was disposed off in a bio‐hazard bag (yellow bag).

### 2.7 Other support

Arrangements for lodging were also made for the patient and two caregivers when needed. During the whole period of staying, local transport was also arranged from lodgement facility to hospital and vice versa. Cankids has seven dedicated homes away from home (HAH) facilities across India, out of which two are in Delhi. These facilities are equipped with cooking facility, and have opportunities for learning, entertainment, celebrations, and therapeutic activities.

The nutritionist, teachers, and psychologists were involved in the holistic care and extended their specialized services to the patients and family members during the stay in hospital care. The nutritionists assessed the dietary need and made a diet plan with the consensus of treating doctors. The teachers actively engaged the patients and made available the customized learning materials, while psychologists assessed the psychosocial need of the patients and family members and counseled them accordingly.

### 2.8 Data management and analysis

The socio‐demographic and required clinical information on these patients was gathered from the hospital records. The data about transport, support staff involved, gap financing for quality drugs, diagnostics, and standard treatment, and so on were collected from the management information system‐vCAN (virtual Cankids Assistance Network) of the Cankids. Cankids has its own cloud‐based integrated customer relationship management platform, developed by Salesforce for data entry, single shared view, and analysis.

The outcome of the interest was avoiding death, abandonment, and relapse due to treatment delay. The data were analyzed by the Cankids management team.

## 3 RESULTS

### 3.1 Hospitals and patients

Five hospitals in North and East India that had been declared as COVID hospitals approached Cankids for shared care. (Table 1) The total number of patients registered for diagnosis, treatment, follow‐up, and post‐treatment in all five hospitals of origin was 190. Of these 58 patients needed active cancer care treatment in in‐patient or daycare and were required to shift to destination hospital. While three families were not ready to shift to another hospital due to apprehension of COVID‐19, 55 (95%) families gave their consent to shift. 86.2% of these patients are alive and treatment abandonment and relapse due to delay in treatment were not observed during the period of shared care. The detail of these patients is given in Table 2.

**TABLE 1:** Distribution of the number of children mobilize to in‐patient department in the different cancer‐treating center during COVID‐19 (*N* = 55)

Hospital of origin (Sector)	Declared COVID hospital		Destination hospital (Sector)	Number of patients
LNJP Hospital, Delhi (Public)	April 2020 till date	A	Max Super Specialty Hospital, Saket, Delhi (Private)	9
		B	Indraprastha Apollo Hospital, Delhi (Private)	1
GTB Hospital, Delhi (Public)	April 2020 till date	A	Max Super Specialty Hospital, Vaishali, Delhi (Private)	4
		B	Max Super Specialty Hospital, Saket, Delhi (Private)	7
		C	Indraprastha Apollo Hospital, Delhi (Private)	5
SSPH, Noida, Uttar Pradesh (Public)	April 2020 till May	A	Max Super Specialty Hospital, Saket, Delhi (Private)	1
		B	Indraprastha Apollo Hospital, Delhi (Private)	2
MCK, West Bengal (Public)	April 2020 till date	A	Upkar Nursing Home, Kolkata, West Bengal (Private)	16
AIIMS, Patna, Bihar (Public)^a^	July 2020 till date	A	Indraprastha Apollo Hospital, Delhi (Private)	6
		B	Paras HMRI Hospital, Patna, Bihar (Private)	1
		C	Buddha Cancer Centre, Patna, Bihar (Private)	3

^a^ Bihar government declare all hospitals to be COVID hospital initially, later government exempted the cancer hospital.

*Note*: LNJP‐Lok Nayak Jai Prakash Narayan Hospital, GTB‐Guru Teg Bahadur, SSPH‐ Super‐specialty Pediatric Hospital, MCK‐Medical College Kolkata, AIIMS‐ All India Institute of Medical Sciences.

**TABLE 2:** | Distribution of the socio‐demographic and clinical characteristics of the beneficiaries of shared care (*N* = 55)

Characteristics	Frequency (%)
Age in Median (IQR)	8 (4–11)
Gender	
Male	37 (67.3)
Female	18 (32.7)
State	
West Bengal	16 (29.1)
Uttar Pradesh	15 (27.3)
Delhi	12 (21.8)
Bihar	11 (20.0)
Haryana	1 (1.8)
Type of family	
Nuclear	41 (74.5)
Joint	9 (16.4)
Not available	5 (9.1)
Family member	
3–5	33 (60.0)
6–9	22 (40.0)
Socio‐economic status by Kuppuswamy scale	
Lower‐middle class	5 (9.1)
Upper‐lower	40 (72.7)
Lower	10 (18.2)
Diagnosis	
Acute lymphoblastic leukemia	46 (83.6)
Other leukemia/lymphoma	4 (7.3)
Other solid tumors	5 (9.1)
Type of assistance	
Chemotherapy & Supportive Care	45 (81.8)
Radiation	6 (10.9)
Diagnostic	3 (5.5)
Surgery	1 (1.8)
Treatment status	
On treatment	54 (98.2)
Palliative care	1 (1.8)
Current status	
Alive	48 (86.2)
Expired^a^	7 (13.8)

^a^ Expired: ‐ On treatment = 6, Palliative care =1.

### 3.2 Social support

Thirty‐four Cankids social support team members including social workers, teachers, dieticians, and child counselors supported by the management staff from Cankids were involved in the process of shifting 555 patients to the destination hospital. They worked closely with the treating team at the origin and destination hospitals. A total of 151 non‐medical support sessions including counseling, the continuum of education, nutritional support, transport, and accommodation were provided. The transportation including within the city and/or to another city in the case was arranged by Cankids through its vehicle, outsourced, and reimbursed if patients arrange in an emergency. There were 291 such trips. The lodging (home away home) arrangement was done by Cankids and all the patients combined stayed a total of 174 days. The total cost of medical facilitation on the diagnostics, drugs, and treatment of the 55 patients was INR 61,61 636 ($ 84 843). The median (IQR) of the facilitation was INR 41765 (IQR 19491‐174 129) INR 6183249 ($ 84129.04). The medical facilitation provided by Cankids on diagnostic, chemotherapy, and supportive care, radiation, and surgery were INR 327712, INR 5073384, INR 649241, and INR 111298, respectively.

## 4 DISCUSSION

The COVID‐19 Pandemic and the national lockdown created the need for finding solutions. The previous experiences helped us to make a sustainable plan during the lockdown and Cankids start working to identify the possible hurdles. After being designated as COVID centers, five hospitals asked Cankids to leverage its vast network for a continuum of care of patients with childhood cancer. Cankids extended not only medical but other holistic support also. As a result, of those patients who needed active treatment, 95% were able to continue this allowing them to have a chance of cure and care.

Several hospitals in India have taken initiatives to provide ongoing treatment to their patients. Department of Pediatrics, Postgraduate Institute of Medical Education and Research, Chandigarh India, managed the pediatric cancer patients through telecommunication and made a WhatsApp group with 35 pediatricians in the vicinity of the patients and with 200 families to instruct. They managed febrile neutropenia and parenteral chemotherapy including intrathecal methotrexate with the local pediatrician.^9^ Pediatric Oncology Division, All India Institute of Medical Sciences, New Delhi, advised the patients to continue oral chemotherapy and defer the intensive chemotherapy. They create a support group named “Sambhav” and communicated through telephone, helplines, and emails with the patients. They took help from the non‐government organization for antibiotic administration, accommodation, coordination with the local hospital, transferring medicines to distant patients, and transportation facility within and across states.^10^ A similar example of a teleconsulting facility practiced in Pediatric Oncology Division, Tata Memorial Hospital, Mumbai. They prevent unwarranted visits of patients to the hospital premises were on follow‐up.^11^

Our model is different from the above‐mentioned examples and works at the inter‐hospital level rather than the intra‐hospital level. It complements the above‐mentioned hospital‐led initiatives in childhood cancer in India. As soon as we received a request from the hospital of origin, we communicated to the destination hospital within the city to accommodate the patients. We negotiated the price with the private hospitals to continue treatment at an affordable cost. Cankids extend support for financing, transportation, accommodation, an arrangement of food, and liaising with both the hospital of origin and destination hospital. The treatment protocol was continued as decided by the treating doctor in the hospital of origin. Treatment abandonment and relapse due to delay in treatment were not observed during the period of shared care. It could be due to timely intervention to the continuity of the treatment protocol. During and after the shared care process, Cankids has been collecting feedback from the families to identify the challenges faced by patients and families in this transition. The data of feedback from families will help us to improve the service and prepare for the future.

Cancer itself has a significant psychosocial effect on patients and their families, and the pandemic worsened the situation because of challenges to accessing care as well as unforeseen problems like loss of employment/income.^12^ The role of civil society is important in this pandemic time and Cankids come forward with gap financing and arrange funds for emergency medical assistance, drugs, and other treatment interventions. The impact of the shared care provided comfort, rapport, and trust with patients/ caregivers, continuity of care, patient‐centric care processes, coordination between hospital of origin and destination hospital, motivation, and adherence to the treatment.

## 5 CONCLUSION

Access to services constitutes a major barrier to cancer diagnosis and treatment for the majority of children in LMIC. The shared care experience during COVID‐19 time provides an opportunity for close coordination between treating hospitals, patients, and parents. The patients in need received all the benefits of specialist intervention combined with the continuity of care. Learnings from this experience need to continue and be implemented even after the pandemic is over. This will help to reduce underdiagnosis, abandonment, and low survival, also relieve some of the financial and logistical constraints to continuing treatment.

## CONFLICT OF INTEREST

The authors declare that they have no conflict of interest.

## AUTHOR CONTRIBUTIONS

**Amita Mahajan:** Conceptualization; data curation; formal analysis; methodology; supervision; writing‐original draft; writing‐review & editing. **Ramandeep Arora:** Conceptualization; data curation; formal analysis; methodology; supervision; writing‐original draft; writing‐review & editing. **Puneet Sahi:** Data curation; validation; writing‐review & editing. **Sunil Gomber:** Data curation; validation; writing‐review & editing. **Nita Radhakrishnan:** Data curation; validation; writing‐review & editing. **Basab Bagchi:** Data curation; validation; writing‐review & editing. **Prachi Jain:** Data curation; validation; writing‐review & editing. **Arvind Kumar:** Data curation; validation; writing‐review & editing. **Avinash Singh:** Data curation; validation; writing‐review & editing. **Haresh Gupta:** Data curation; project administration; resources; supervision; validation; writing‐review & editing. **Sonal Sharma:** Project administration; resources; validation; writing‐review & editing. **Nasim Ahamad:** Project administration; resources; validation; writing‐review & editing. **Poonam Bagai:** Conceptualization; project administration; resources; supervision; writing‐review & editing. **Arvind Kumar:** Conceptualization; data curation; formal analysis; methodology; project administration; validation; writing‐original draft; writing‐review & editing.

## ETHICS STATEMENT

No ethics approval was taken as this was not part of a research study or intervention but the transition of necessary services to ensure the quality of treatment did not decline. No human subject data are involved; therefore, no patient consent was required.

## Data Availability

The data that support the findings of this study are available from the corresponding author upon reasonable request.
